# CP110 and CEP135 Localize Near the Proximal Centriolar Remnants of Mice Spermatozoa

**DOI:** 10.17912/micropub.biology.001083

**Published:** 2024-01-29

**Authors:** Abirami Subbiah, Drew L. Caswell, Katerina Turner, Ankit Jaiswal, Tomer Avidor-Reiss

**Affiliations:** 1 University of Toledo, Toledo, Ohio, United States

## Abstract

Centrioles form centrosomes that organize microtubules, assist in cell structure, and nucleate cilia that provide motility and sensation. Within the sperm, the centrosome consists of two centrioles (proximal and distal centriole) and a pericentriolar material known as the striated column and capitulum. The distal centriole nucleates the flagellum. Mice spermatozoa, unlike other mammal spermatozoa (e.g., human and bovine), have no ultra-structurally recognizable centrioles, but their neck has the centriolar proteins POC1B and FAM161A, suggesting mice spermatozoa have remnant centrioles. Here, we examine whether other centriolar proteins, CP110 and CEP135, found in the human and bovine spermatozoa centrioles are also found in the mouse spermatozoa neck. CP110 is a tip protein controlling ciliogenesis, and CEP135 is a centriole-specific structural protein in the centriole base of canonical centrioles found in most cell types. Here, we report that CP110 and CEP135 were both located in the mice spermatozoa neck around the proximal centriolar remnants labeled by POC1B, increasing the number of centriolar proteins found in the mice spermatozoa neck, further supporting the hypothesis that a remnant proximal centriole is present in mice.

**Figure 1. CP110 and CEP135 Localize Near the Proximal Centriolar Remnants of Mice Spermatozoa.**
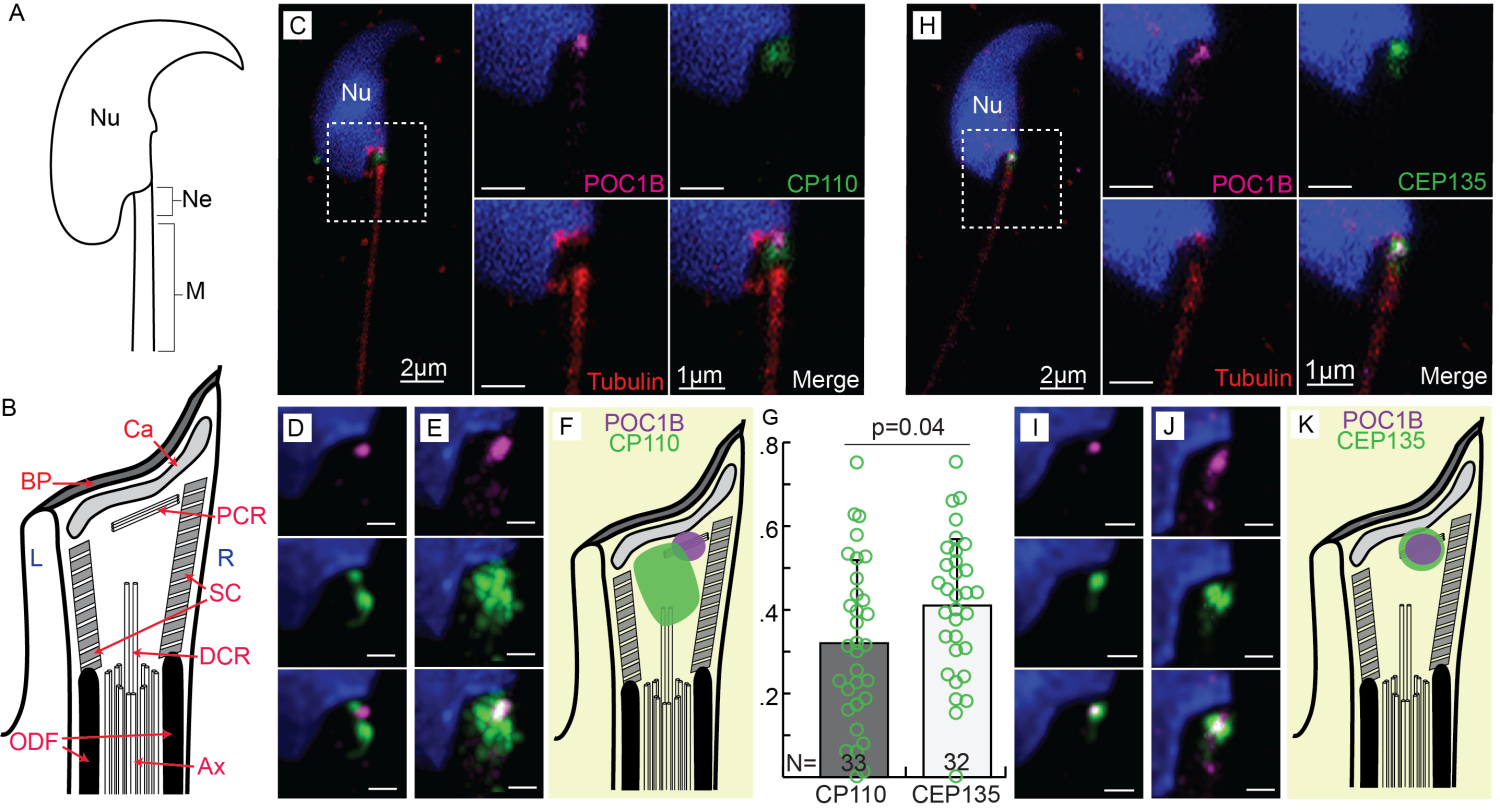
**A)** Drawing of a generic murine sperm including head with the nucleus ( **Nu** ), neck ( **Ne** ), and midpiece ( **M** ). Note the attachment of the neck to the head: the implantation fossa takes the shape of a groove. **B)** The mouse sperm neck contains the basal plate ( **BP** ), capitulum ( **Ca** ), microtubule remnants of the proximal centriole ( **PCR** ) and distal centriole ( **DCR** ), axoneme ( **Ax** ), striated column ( **SC** ), and outer dense fibers ( **ODF** ). Sperm left side ( **L** ) and right side ( **R** ). **C, H)** Each panel contains two groups of images from left to right: a medium magnification image (scale bar 2 µm) and four high magnification images (scale bar 1 µm) from the same spermatozoa. Antibodies against POC1B (magenta) are labeled near the proximal centriolar remnant of the mouse spermatozoa. Hoechst labels the nucleus (blue). Anti-tubulin (red) labels the axoneme and below the nucleus. Anti-CP110 ( **C** ) and anti-CEP135 ( **H** ) antibodies are depicted in green. **D, I)** Panels with three HyVolution images highlighting the location of CP110 ( **D** ) or CEP135 ( **I** ) relative to POC1B labeling from a single spermatozoon. **E, J)** Panels with three images that were made by overlaying ten different HyVolution images with POC1B as a reference point indicating the relative localization of CP110 ( **E** ) or CEP135 ( **J** ) within the neck (scale bar 0.5 µm). **F, K)** Drawings depicting the localization of CP110 or CEP135 relative to structures within the neck. **G)** Graph representing the colocalization of POC1B with CP110 or CEP135. The average colocalization of CEP135 and POC1B was 0.41±0.16, and the average colocalization of CP110 and POC1B was 0.32±0.19 (p=0.04).

## Description

Centrioles are essential in cell biology, forming the centrosome and nucleating cilia. Centrosomes organize microtubules during interphase and cell division, providing cytoskeletal structure to the cell (Bornens 2002; Satir and Christensen 2007; Rusan and Rogers 2009; Joukov and De Nicolo 2019) . Cilia are membrane-bound, microtubule-based protrusions that emanate from the distal end of a centriole and serve as a sensory organelle that a cell uses for signal transduction and reception, as well as cell motility (Mill *et al.* 2023). A standard centrosome or cilia consists of two barrel-shaped centrioles that differ slightly in structure and function (Uzbekov *et al.* 2012). In a centrosome, the centrioles are surrounded by a network of scaffolding proteins known as pericentriolar material (Schatten and Sun 2009) .

Mammalian spermatozoa have a common organization of head, neck, and tail ( **Fig 1A);** however, they differ in many details in various species. Most mammals have a pear or paddle-like sperm head (Gu *et al.* 2019). The neck is usually attached to the center of the nucleus base (Khanal *et al.* , 2023) but has a left-right asymmetry (Khanal *et al.* , 2021). The nucleus caudal pole has a shallow depression called the implantation fossa, which houses the sperm neck attachment. In contrast, the mouse spermatozoa head has a falciform shape, and the tail is attached at the side of the nucleus base, resulting in a deep implantation fossa that takes the shape of a groove ( **Fig 1A** ) (Fawcett 1975; Wyrobek *et al.* 1976; Hoyer-Fender 2022). The offset tail attachment in mice is suspected to have evolved in parallel with changes in the female reproductive tract (Tung and Suarez 2021) and the changes in spermatozoa centrioles (Khanal *et al.* 2023).

Within sperm cells, centrioles are essential for the tail formation, linking the sperm tail to the head and creating the microtubule organization center within the zygote (Avidor-Reiss *et al.* , 2020; Avidor-Reiss and Uzbekov, 2023; Xie *et al.* 2023). Centrioles have evolved into many diverse structures in the sperm cells of both invertebrates and vertebrates, presumed to be due to the selective pressure of sperm competition associated with internal fertilization (Fishman *et al.* 2017; Fisher *et al.* 2022; Turner *et al.* 2022a). In most mammals, spermatozoa have one structurally canonical, barrel-shaped, proximal centriole that resides just below the nucleus and one atypical, funnel-shaped distal centriole that connects to the axoneme (Fishman *et al.* 2018). These two centrioles, the striated column and capitulum, are dynamic links between the tail and head (Avidor-Reiss and Fishman 2019; Khanal *et al.* 2021). Unlike non-murine mammals, the spermatozoa of mice appear to have no recognizable centrioles, but they do have a striated column and capitulum ( **Fig 1B** ).

During spermiogenesis, mouse sperm centrioles undergo centrosome reduction, a more dramatic remodeling process than other mammals; it includes three stages: microtubule nucleation loss-of-function, the scattering of γ-Tubulin, and the degradation of centrioles (Manandhar *et al.* 1998). Currently, the dogma is that mice spermatids initially contain two centrioles, but after undergoing spermatogenesis, the spermatozoan centrioles completely degenerate (Schatten *et al.* 1986; Manandhar *et al.* 1998; Manandhar *et al.* 2005). However, Leung and colleagues (2021) have shown by cryo-electron tomography that a few centriolar microtubules remained in the mouse spermatozoa neck (Leung *et al.* 2021). Although there are no recognizable intact centriolar structures, there was still localization of the centriolar proteins POC1B at the proximal centriolar remnant and FAM161A at the distal centriolar remnant of the spermatozoa. Thus, the centriole degradation of mice spermatozoa may be a more severe form of remodeling, eliminating the centriole structure, but it may maintain some centriole proteins (Khanal *et al.* , 2023).

To understand the localization of other centriolar proteins in mice spermatozoa, we used confocal (~250 nm X-Y resolution) and HyVolution (~80 nm X-Y resolution) microscopy to study centriolar protein localization. We used tubulin to label the tail and Hoechst to label the head. Since spermatozoa are asymmetrical, it was pertinent to orient the head consistently. We oriented the sperm with the groove as the bottom right of the nucleus since the proximal centriole tip grows in this direction ( **Fig 1B** ) (Garanina *et al.* 2019) as we have done in previous studies (Khanal *et al.* 2023; Turner *et al.* 2023).

To localize the centriolar protein, we studied their labeling relative to the known mice spermatozoon centriolar protein POC1B. POC1B is a protein found in the centriole lumen and is essential for the structural integrity of centrioles and cilia (Pearson *et al.* , 2009). Loss-of-function mutations in POC1B lead to incorrect centriole formation and maintenance (Venoux *et al.* 2013; Zhang *et al.* 2015), abnormal spermatozoa morphology and movement, and infertility (Hua *et al.* 2023). POC1B specifically labels the proximal and distal centrioles in human and bovine spermatozoa, as well as the atypical centriole in fruit fly spermatozoa (Khire *et al.* 2016; Fishman *et al.* 2018; Jo *et al.* 2019). In mice, anti-POC1B antibody labels a focus in the neck just below the nucleus, the presumed site of the proximal centriole remnant (Khanal *et al.* 2023). As expected, we also observed an anti-POC1B antibody labeled directly under the nucleus (99% of spermatozoa, N=123). However, we also saw in a few cases that POC1B labeled a second focus above the axoneme (20% of spermatozoa, N=123). This second site may be the distal centriolar remnant.

Centriolar coiled-coil protein 110 (CP110) is a capping protein that localizes at the distal ends of the mother and daughter centrioles (Kleylein-Sohn *et al.* 2007). CP110 primarily acts as a critical suppressor of ciliogenesis by forming an intricate complex with several other proteins to physically block microtubules from growing into a cilium (Tsang *et al.* 2008; Gerdes *et al.* 2009; Otto and Hoyer-Fender 2023). However, the loss-of-function of CP110 resulted in incomplete centriole docking at the plasma membrane and a lack of cilia formation (Yadav *et al.* 2016).

Recently, we discovered that CP110 antibodies labeled the base of the proximal centriole and distal centriole along with part of the pericentriolar material in human and bovine spermatozoa (Turner *et al.* 2023). Labeling occurs in humans at the proximal centriole, the capitulum, and the striated column. This surprising out-of-the-centriole labeling lead us to hypothesize that CP110 re-localizes during the centriolar remodeling process as part of the striated column and capitulum formation.

Here, we studied CP110 localization in mice spermatozoa using a rabbit polyclonal antibody against CP110’s amino acids 1-337 (12780-1-AP). The anti-CP110 antibody labeled the area around the POC1B-labeled proximal centriole remnant between the Hoechst-labeled nucleus and tubulin-stained axoneme (73%, 22/30) ( **Fig 1C** ). Results from HyVolution imaging showed similar results (87%, 29/33) ( **Fig 1D** ). In both confocal and HyVolution images, the CP110 localization showed high variance and appeared labeled in different locations around the POC1B-labeled proximal centriole remnant. Overlays of ten HyVolution images using POC1B-labelling as a reference point showed the various localizations around the proximal centriole remnant ( **Fig 1E** ). CP110 and POC1B had a colocalization rate of 32±19% ( **Fig 1G** ).

Centriolar protein 135 (CEP135) is a structural protein with microtubule binding affinity essential for the centriole's formation (Ohta *et al.* 2002). CEP135 is part of the scaffolding, keeping the centriole microtubule wall in place (Lin *et al.* 2013). In human cells, CEP135 localizes to the centriole cartwheel, a structure found at the centriole base, and mutations in this protein are associated with abnormal centrosomes (Tian *et al.* , 2021; Chu and Gruss, 2022). Mutations in human CEP135 result in defects in the proximal centriole and spermatozoa tail morphology (Sha *et al.* 2017). More precisely, CEP135 labeled the proximal centriole base, the distal centriole base, and the striated columns to the right of the distal centriole in human and bovine spermatozoa (Sha *et al.* 2017; Turner *et al.* 2023).

Here, we studied CEP135 localization in mice spermatozoa using a polyclonal rabbit antibody (24428-1-AP) against amino acids 1- 233 of CEP135. In the confocal images, the anti-CEP135 labeling colocalized with the POC1B labeling but also distributed over a slightly larger region (97% of spermatozoa, 29/30) ( **Fig 1H** ). HyVolution images found colocalization with POC1B (80% of spermatozoa, 24/30) and localization 0.25 μm around the POC1B (20% of spermatozoa, 6/30) ( **Fig 1I** ). CEP135 partly colocalized with the POC1B labeling of the proximal centriole remnant and had a colocalization rate of 41% ± 12% **(Fig 1G)** . Three overlays of HyVolution images, each using POC1B-labeling as a reference point, supported this localization pattern ( **Fig 1J** ).

Overall, we found that the centriolar proteins CP110 and CEP135 are located near the proximal centriole remnant marker POC1B of mice, supporting the hypothesis that mice have a remnant proximal centriole and perhaps some residual function.

## Methods

**Immunofluorescence**

Preparation: Epididymis spermatozoa were isolated from sexually mature captive-bred house mice ( *Mus musculus* used in a research lab setting). Euthanized mice were dissected, and the epididymis was further isolated. The epididymis was moved to a Petri dish containing sterile phosphate-buffered saline (PBS), where it was dissected into small pieces using surgical scissors. The plate was then incubated in a CO _2_ incubator at 37°C for 1 hour to allow sperm to swim out of the tissue. PBS containing swimming sperm was collected in a tube and centrifuged at 1000 × *g* for 8 minutes. The supernatant was discarded, and the pellet was resuspended in an appropriate volume (100–200 μL).

Spermatozoa was cryogenically preserved by adding 1 volume of GALAP to 1 volume of sperm. Then, two volumes of freezing diluter (3.5M DMSO, 0.1M sucrose in GALAP) were added dropwise while mixing thoroughly. Sperm was then aspirated into a straw, sealed, and placed on ice for 45 minutes. Straws were placed 5 cm above liquid nitrogen in the vapors for 15 minutes. After, they were submerged into the liquid nitrogen and transferred to a liquid nitrogen tank for storage until use.

When ready to use, the straw was plunged into 37°C water for approximately 2 minutes. About 30 μL of the sperm sample was added and spread evenly across the bottom half of each slide and set out to air dry overnight. The sperm slides were then stored in the fridge at 4°C until needed.

Staining: The slides were placed on a hot plate at 45°C for 5-10 minutes to dry and then placed in a pre-chilled Coplin jar with methanol at –20°C for two minutes to fix the sperm. Slides were then put into a PBS wash for 5 minutes, followed by permeabilization with PBST for 45 minutes. The slides were placed into PBSTB for 45 minutes for blocking. Primary antibodies were diluted in PBSTB, the solution was added, and slides were incubated in a humidified chamber at 4°C overnight. The slides were washed in PBST three times for five minutes each. Secondary antibodies and Hoechst were diluted in PBSTB, then added to the slide, and incubated for two hours at room temperature in a humidified chamber. The slides were washed three times in PBST and three times in PBS for five minutes each. A drop of VectaShield mounting media and a cover slide were added. The slides were sealed with nail polish and stored in a fridge at 4°C until imaging.

**Imaging**

Confocal Microscopy: Slides were visualized using a Leica SP8 confocal microscope in BrightR mode with an HC PL APO CS2 63x/1.40 OIL lens, 3x zoom factor, 100% gain, 1024 × 1024 pixels (62 μM x 62 μM) format with a line averaging of 3, frame accumulation of 2, and a 90° rotation. To collect the fluorescence signals, three sequences were used. The first sequence consisted of DNA and phase-like images. The second sequence had CEP135 or CP110 and POC1B images, while the third sequence had tubulin images. A 410 nm laser was used to get the DNA staining. The absorption spectrum was set to collect over 425–478 nm via the HyD1 detector and was color-coded to blue. The fluoro-turret was set to Scan-PH for the phase-like image, and PMT Trans was turned ON with a gain of 280. To get CEP135 or CP110 staining via ALEXA 488, the 488 nm laser was activated at 3.5%. The absorption spectrum was set to 508-543 nm via the HyD3 detector, color-coded to green. For the POC1B staining via ALEXA 647, the 633 nm laser was activated at 4%. The absorption spectrum was set to 650–690 nm via a HyD4 detector and was color-coded to magenta. To get tubulin via ALEXA 555, the 561 nm laser was activated at 3%. The absorption spectrum was set to 566-623 nm via the HyD4 detector and was color-coded to red. 9-16 Z-sections of 0.3 μM thickness from the top to the bottom of the sperm were collected.

HyVolution: Similar conditions were used, except the zoom factor was set to 6x, the standard mode was used instead of BrightR, the gain was set to 15%, and no phase images were taken. DNA images were produced in the first sequence, CEP135 or CP110, POC1B images in the second sequence, and tubulin in the third sequence. Images were deconvoluted using HyVolution II from Leica Microsystems. The SVI Huygens Essential program was used with the typical strategy, no auto-cropping, and the mounting media refractive index was set for Vectashield (1.457).

**Image Preparation and Analysis**

Confocal: TIF images generated by the confocal microscope were imported into Adobe Photoshop. Photoshop was used to increase intensity and rotate the images of the sperm so that the head was towards the top and the tail was towards the bottom of the image. The image was reflected when needed so that the groove of the mouse's sperm head was oriented to the right side. A 500 x 1000-pixel low-magnification image included the head, neck, and a portion of the tail. A medium magnification of 150 x 300-pixel image was created to show the head, neck, and centrioles. Four high-magnification 75 x 75-pixel images were made to focus on the sperm neck as well as each antibody. The images were adjusted to 1 inch x 2 inch for low and medium magnification and 1 inch x 1 inch for high magnification. These images were then exported into an Adobe Illustrator file, arranged and labeled with antibody names and a scale bar for each image.

HyVolution: TIF images were generated and imported into Adobe Photoshop as above. Each HyVolution image was made as 75 x 75 pixels for only the POC1B and experimental biomarker and given dimensions of 0.667 in x 0.667 in. 10 HyVolution images from each experiment data was used to create an overlay image, using POC1B staining as a reference point. These images were given dimensions 0.667 in x 0.667 in.

Three slides were stained for each experimental biomarker, and 10-15 sperm from each slide were imaged using the confocal in BrightR, and another ten sperm from each slide were imaged using HyVolution.

**Colocalization Analysis**

Colocalization between POC1B and CP110 or CEP135 was quantified using the “Colocalization Report” tool in the Leica LAS-X program. A rectangle of 1 μm x 2 μm was drawn under the base of the nucleus to capture the area of interest. Relative overlap of staining between POC1B and either CP110 or CEP135 was determined within the box for each HyVolution image.

**Antibody Validation**

The validity of CP110 and CEP135 was tested by staining mice 3T3 fibroblast cells with POC1B, γ-tubulin, and tubulin. POC1B and γ-tubulin are known centriolar biomarkers that will label the centrioles in 3T3 cells. Labeling the cell with a known centriolar and experimental biomarker can confirm whether the antibody is labeling the centriole. The 3T3 cells were grown on a coverslip kept in six-well plates during the immunostaining procedure. The cells were washed in PBS for 5 minutes and then covered in 0.3% PBST (with 250 mL of PBS and 750 μL of Triton) for 45 minutes to permeabilize. The cells were then covered with 5% nonfat dry milk with PBST (5 mL of PBST and 0.25 g of nonfat dry milk powder) for 45 minutes for blocking. The primary antibody solution was added, and the coverslips were incubated in a humidified chamber at 4°C overnight. The cells were washed in PBST three times for five minutes each. Secondary antibodies and Hoechst were then added to the coverslips and incubated for two hours at room temperature in a humidified chamber. The cells were washed three times in PBST for five minutes and three times in PBS for five minutes. A drop of VectaShield mounting media and a cover slide were added. The slides were sealed with nail polish and stored in a fridge at 4°C until imaging. CEP135 was added with POC1B, while CP110 was added with γ-tubulin.

## Reagents

**Primary Antibodies**

Polyclonal anti-POC1B made in mice (Thermo Fisher Scientific, H00282809-B01P) was raised against a full-length human POC1B. The antibody specificity was shown previously in (Turner *et al.* 2022b) (Diluted 1:300).

Sheep anti-Tubulin (Cytoskeleton, Inc. ATN02) specificity was demonstrated in (Piroli *et al.* 2014; Lobert *et al.* 2022) (Diluted 1:500).

Polyclonal Rabbit anti-CEP135 (Thermo Fisher Scientific, 24428-1-AP) was raised against amino acids 1-233 of CEP135, and specificity was demonstrated in (Gupta *et al.* 2020) (Diluted 1:200).

Polyclonal rabbit anti-CP110 (Thermo Fisher Scientific, 12780-1-AP) was raised against amino acids 1-377 of CP110. (Diluted 1:200).

**Secondary Antibodies**

Anti-Mouse Alexa 488 made in Donkey (Thermo Fisher Scientific, A-21436) (Diluted 1:400).

Anti-Sheep Alexa 555 made in Donkey (Thermo Fisher Scientific, A-21436) (Diluted 1:500).

Anti-Rabbit Dylight 650 made in Donkey (Thermo Fisher Scientific, SA5-10041) (Diluted 1:300).

Anti-Mouse Alexa 647 made in Donkey (Thermo Fisher Scientific, 715-605-150) (Diluted 1:300).

Anti-Rabbit Alexa 488 made in Donkey (Thermo Fisher Scientific, 711-545-152) (Diluted 1:400).

**Solutions**

Washing solution: phosphate-buffered saline (PBS)

Permeabilization Buffer (PBST): is made of PBS with 0.3% Triton X-100 (Sigma Aldrich, 9002-93-1)

Blocking Solution (PBSTB): Is made of PBST with 1% BSA (Bovine Serum Albumin) (CHEM-IMPEX INT’L, 00535)

Fixation media: Methanol (-20°C) (Fisher Chemical, A412P-4)

Mounting media: Fluoroshield with DAPI (Sigma-Aldrich, F6057-20ML)

Hoechst Stain: Thermo Fisher Scientific, H1399 (10 mg/mL) (Diluted 1 to 1000)

Slide sealing media: Nail polish, EMS Diasum, 72180

Preservation media: GALAP (IMV Technologies, 028656, 500 mL)

**Materials**

Slides and Cover Slips: Glass slide (Azer Scientific, EMS200A+), glass coverslip (VWR, 48366-205)

Glass Coplin jars: Research Products International Corp. Cat. # 50-212-281

## References

[R1] Avidor-Reiss T, Carr A, Fishman EL (2020). The sperm centrioles.. Mol Cell Endocrinol.

[R2] Avidor-Reiss T, Fishman EL (2019). It takes two (centrioles) to tango.. Reproduction.

[R3] Avidor-Reiss T, Uzbekov R (2023). Revisiting the mystery of centrioles at the beginning of mammalian embryogenesis.. J Assist Reprod Genet.

[R4] Bornens M (2002). Centrosome composition and microtubule anchoring mechanisms.. Curr Opin Cell Biol.

[R5] Chu Z, Gruss OJ (2022). Mitotic Maturation Compensates for Premature Centrosome Splitting and PCM Loss in Human cep135 Knockout Cells.. Cells.

[R6] Fawcett DW (1975). The mammalian spermatozoon.. Dev Biol.

[R7] Fisher HS, Roldan ERS, Avidor-Reiss T, Rowe M (2022). On the Origin and Evolution of Sperm Cells.. Cells.

[R8] Fishman EL, Jo K, Ha A, Royfman R, Zinn A, Krishnamurthy M, Avidor-Reiss T (2017). Atypical centrioles are present in Tribolium sperm.. Open Biol.

[R9] Fishman EL, Jo K, Nguyen QPH, Kong D, Royfman R, Cekic AR, Khanal S, Miller AL, Simerly C, Schatten G, Loncarek J, Mennella V, Avidor-Reiss T (2018). Author Correction: A novel atypical sperm centriole is functional during human fertilization.. Nat Commun.

[R10] Garanina AS, Alieva IB, Bragina EE, Blanchard E, Arbeille B, Guerif F, Uzbekova S, Uzbekov RE (2019). The Centriolar Adjunct⁻Appearance and Disassembly in Spermiogenesis and the Potential Impact on Fertility.. Cells.

[R11] Gerdes JM, Davis EE, Katsanis N (2009). The vertebrate primary cilium in development, homeostasis, and disease.. Cell.

[R12] Gu NH, Zhao WL, Wang GS, Sun F (2019). Comparative analysis of mammalian sperm ultrastructure reveals relationships between sperm morphology, mitochondrial functions and motility.. Reprod Biol Endocrinol.

[R13] Gupta H, Rajeev R, Sasmal R, Radhakrishnan RM, Anand U, Chandran H, Aparna NR, Agasti S, Manna TK (2020). SAS-6 Association with γ-Tubulin Ring Complex Is Required for Centriole Duplication in Human Cells.. Curr Biol.

[R14] Hoyer-Fender S (2022). Development of the Connecting Piece in ODF1-Deficient Mouse Spermatids.. Int J Mol Sci.

[R15] Hua Juan, Xu Bo, Liu Wenjing, Shi JingTian, Jiang Hui, Zha XiaoJun, Zhang Xiansheng, Wan Yangyang (2023). Homozygous frameshift variant in POC1B causes male infertility with oligoasthenoteratozoospermia in human and mice. Human Molecular Genetics.

[R16] Jo KH, Jaiswal A, Khanal S, Fishman EL, Curry AN, Avidor-Reiss T (2019). Poc1B and Sas-6 Function Together during the Atypical Centriole Formation in Drosophila melanogaster.. Cells.

[R17] Joukov V, De Nicolo A (2019). The Centrosome and the Primary Cilium: The Yin and Yang of a Hybrid Organelle.. Cells.

[R18] Khanal Sushil, Jaiswal Ankit, Chowdanayaka Rajanikanth, Puente Nahshon, Turner Katerina, Assefa Kebron Yeshitela, Nawras Mohamad, Back Ezekiel David, Royfman Abigail, Burkett James P., Cheong Soon Hon, Fisher Heidi S., Sindhwani Puneet, Gray John, Ramachandra Nallur Basappa, Avidor-Reiss Tomer (2024). The evolution of centriole degradation in mouse sperm. Nature Communications.

[R19] Khanal S, Leung MR, Royfman A, Fishman EL, Saltzman B, Bloomfield-Gadêlha H, Zeev-Ben-Mordehai T, Avidor-Reiss T (2021). A dynamic basal complex modulates mammalian sperm movement.. Nat Commun.

[R20] Khire A, Jo KH, Kong D, Akhshi T, Blachon S, Cekic AR, Hynek S, Ha A, Loncarek J, Mennella V, Avidor-Reiss T (2016). Centriole Remodeling during Spermiogenesis in Drosophila.. Curr Biol.

[R21] Kleylein-Sohn J, Westendorf J, Le Clech M, Habedanck R, Stierhof YD, Nigg EA (2007). Plk4-induced centriole biogenesis in human cells.. Dev Cell.

[R22] Leung MR, Roelofs MC, Ravi RT, Maitan P, Henning H, Zhang M, Bromfield EG, Howes SC, Gadella BM, Bloomfield-Gadêlha H, Zeev-Ben-Mordehai T (2021). The multi-scale architecture of mammalian sperm flagella and implications for ciliary motility.. EMBO J.

[R23] Lin YC, Chang CW, Hsu WB, Tang CJ, Lin YN, Chou EJ, Wu CT, Tang TK (2013). Human microcephaly protein CEP135 binds to hSAS-6 and CPAP, and is required for centriole assembly.. EMBO J.

[R24] Lobert VH, Skardal ML, Malerød L, Simensen JE, Algra HA, Andersen AN, Fleischer T, Enserink HA, Liestøl K, Heath JK, Rusten TE, Stenmark HA (2022). PHLPP1 regulates CFTR activity and lumen expansion through AMPK.. Development.

[R25] Manandhar G, Schatten H, Sutovsky P (2004). Centrosome reduction during gametogenesis and its significance.. Biol Reprod.

[R26] Manandhar G, Sutovsky P, Joshi HC, Stearns T, Schatten G (1998). Centrosome reduction during mouse spermiogenesis.. Dev Biol.

[R27] Mill P, Christensen ST, Pedersen LB (2023). Primary cilia as dynamic and diverse signalling hubs in development and disease.. Nat Rev Genet.

[R28] Ohta T, Essner R, Ryu JH, Palazzo RE, Uetake Y, Kuriyama R (2002). Characterization of Cep135, a novel coiled-coil centrosomal protein involved in microtubule organization in mammalian cells.. J Cell Biol.

[R29] Otto M, Hoyer-Fender S (2023). ODF2 Negatively Regulates CP110 Levels at the Centrioles/Basal Bodies to Control the Biogenesis of Primary Cilia.. Cells.

[R30] Pearson CG, Osborn DP, Giddings TH Jr, Beales PL, Winey M (2009). Basal body stability and ciliogenesis requires the conserved component Poc1.. J Cell Biol.

[R31] Piroli GG, Manuel AM, Walla MD, Jepson MJ, Brock JW, Rajesh MP, Tanis RM, Cotham WE, Frizzell N (2014). Identification of protein succination as a novel modification of tubulin.. Biochem J.

[R32] Rusan NM, Rogers GC (2009). Centrosome function: sometimes less is more.. Traffic.

[R33] Satir P, Christensen ST (2007). Overview of structure and function of mammalian cilia.. Annu Rev Physiol.

[R34] Schatten H, Schatten G, Mazia D, Balczon R, Simerly C (1986). Behavior of centrosomes during fertilization and cell division in mouse oocytes and in sea urchin eggs.. Proc Natl Acad Sci U S A.

[R35] Schatten H, Sun QY (2009). The role of centrosomes in mammalian fertilization and its significance for ICSI.. Mol Hum Reprod.

[R36] Sha Yan-Wei, Xu Xiaohui, Mei Li-Bin, Li Ping, Su Zhi-Ying, He Xiao-Qin, Li Lin (2017). A homozygous CEP135 mutation is associated with multiple morphological abnormalities of the sperm flagella (MMAF). Gene.

[R37] Tian Y, Wei C, He J, Yan Y, Pang N, Fang X, Liang X, Fu J. 2021. Superresolution characterization of core centriole architecture. J Cell Biol 220(4). 10.1083/jcb.202005103 PMC7863704 33533934

[R38] Tsang William Y., Bossard Carine, Khanna Hemant, Peränen Johan, Swaroop Anand, Malhotra Vivek, Dynlacht Brian David (2008). CP110 Suppresses Primary Cilia Formation through Its Interaction with CEP290, a Protein Deficient in Human Ciliary Disease. Developmental Cell.

[R39] Tung CK, Suarez SS (2021). Co-Adaptation of Physical Attributes of the Mammalian Female Reproductive Tract and Sperm to Facilitate Fertilization.. Cells.

[R40] Turner K, Solanki N, Salouha HO, Avidor-Reiss T (2022). Atypical Centriolar Composition Correlates with Internal Fertilization in Fish.. Cells.

[R41] Turner KA, Caswell DL, McGrady BM, Pietras-Allen A, Sedlak J, Nathan C, Parasuraman S, McGann AP, Fazili FM, Bell JR, El Smail KN, Pillai SB, Parry KR, Richardson KP, Ruble K, Jaiswal A, Shah TA, Sindhwani P, Avidor-Reiss T (2023). CP110 and CEP135 localize near the proximal and distal centrioles of cattle and human spermatozoa.. MicroPubl Biol.

[R42] Turner KA, Kluczynski DF, Hefner RJ, Moussa RB, Slogar JN, Thekkethottiyil JB, Prine HD, Crossley ER, Flanagan LJ, LaBoy MM, Moran MB, Boyd TG, Kujawski BA, Ruble K, Pap JM, Jaiswal A, Shah TA, Sindhwani P, Avidor-Reiss T (2022). Tubulin posttranslational modifications modify the atypical spermatozoon centriole.. MicroPubl Biol.

[R43] Uzbekov RE, Maurel DB, Aveline PC, Pallu S, Benhamou CL, Rochefort GY (2012). Centrosome fine ultrastructure of the osteocyte mechanosensitive primary cilium.. Microsc Microanal.

[R44] Venoux M, Tait X, Hames RS, Straatman KR, Woodland HR, Fry AM (2012). Poc1A and Poc1B act together in human cells to ensure centriole integrity.. J Cell Sci.

[R45] Wyrobek AJ, Meistrich ML, Furrer R, Bruce WR (1976). Physical characteristics of mouse sperm nuclei.. Biophys J.

[R46] Xie P, Kocur OM, Cheung S, Ng L, Albertini DF, Rosenwaks Z, Palermo GD (2023). Sperm centriolar factors and genetic defects that can predict pregnancy.. Fertil Steril.

[R47] Yadav SP, Sharma NK, Liu C, Dong L, Li T, Swaroop A (2016). Centrosomal protein CP110 controls maturation of the mother centriole during cilia biogenesis.. Development.

[R48] Zhang C, Zhang Q, Wang F, Liu Q (2015). Knockdown of poc1b causes abnormal photoreceptor sensory cilium and vision impairment in zebrafish.. Biochem Biophys Res Commun.

